# miR-124 promotes proliferation and differentiation of neuronal stem cells through inactivating Notch pathway

**DOI:** 10.1186/s13578-017-0194-y

**Published:** 2017-12-11

**Authors:** Shujie Jiao, Yaling Liu, Yaobing Yao, Junfang Teng

**Affiliations:** Department of Neurology, the First Affiliated Hospital of Zhengzhou University, 1st of Jianshe East Road, Zhengzhou, 450000 China

**Keywords:** miR-124, Proliferation, Differentiation, NSCs, Notch pathway

## Abstract

**Background:**

Neural stem cells (NSCs) are able to differentiate into neurons and astroglia. miRNAs have been demonstrated to be involved in NSC self-renewal, proliferation and differentiation. However, the exact role of miR-124 in the development of NSCs and its underlying mechanism remain to be explored.

**Methods:**

Primary NSCs were isolated from embryos of Wistar rats. Immunocytochemistry was used to stain purified NSCs. miR-124, Delta-like 4 (DLL4), ki-67, Nestin, β-tubulin III, glial fibrillary acidic protein (GFAP), HES1, HEY2, and cyclin D1 (CCND1) expressions were detected by qRT-PCR and western blot. The interaction between miR-124 and DLL4 was confirmed by luciferase reporter assay. Cell proliferation was assessed by MTT assay.

**Results:**

NSCs could self-proliferate and differentiate into neurons and astrocyte. miR-124 was up-regulated and DLL4 was down-regulated during NSC differentiation. DLL4 was identified as a target of miR-124 in NSCs. Ectopic expression of miR-124 or knockdown of DLL4 promoted the proliferation and the formation of NSCs to neurospheres. Moreover, miR-124 overexpression or DLL4 down-regulation improved β-tubulin III expression but decreased GFAP expression in NSCs. Furthermore, enforced expression of DLL4 partially reversed the effects of miR-124 on NSCs proliferation and differentiation. Elevated expression of miR-124 suppressed the expressions of HES1, HEY2, and CCND1 in NSCs, while these effects were attenuated following the enhancement of DLL4 expression.

**Conclusion:**

miR-124 promoted proliferation and differentiation of NSCs through inactivating Notch pathway.

## Background

Neurogenesis in the mammalian central nervous system (CNS) is a complex process that occurs throughout life. During neurogenesis, neural stem cells (NSCs) have the potential to produce three cell sub-lineages, including astrocytes, oligodendrocytes, and neurons [1]. The NSCs are a type of multipotent, self-renewal, and undifferentiated neural progenitor cells residing in the subventricular zone (SVZ) of the adult and developing CNS [2]. NSCs derived from mouse embryonic brains have been extensively studied [3]. Therefore, transplantation of embryonic stem cell (ESC)-derived NSCs has been promulgated as a new potential therapeutic approach for several neurodegenerative disorders and psychiatric disorders including Alzheimer’s disease, Parkinson’s disease, spinal cord injuries and Huntington’s disease [4], highlighting a potential prospection in developing strategy for neural replacement and regeneration in tissue engineering. There is striking evidence demonstrating that proliferation and differentiation of neural stem cells (NSCs) are implicated in the development of normal functional mammalian brain [5, 6]. Therefore, it is crucial to explore ways to improve the proliferation and neuronal differentiation of endogenous NSCs, and identify the underlying molecular mechanisms.

microRNAs (miRNAs), single stranded non-coding transcripts with 22–24 nucleotides in length, could trigger translational repression or mRNA degradation via complementary with the 3′ untranslated region (3′UTR) of target genes. Dysregulation of miRNA has been demonstrated to be linked with many diseases via regulating various biological functions such as cell differentiation, proliferation, apoptosis, migration and metastasis, providing a perspective on achieving safe and targeted delivery of miRNA therapeutics [7]. Recently, increasing studies have highlighted the involvement of miRNAs in neural stem cell self-renewal, development, proliferation and differentiation via control of different stem cell regulators [8–10]. miR-124 is one of the most abundant and the best characterized miRNA specifically expressed in the adult brain [11]. miR-124 expression was discovered to be overexpressed in neuronal differentiation, both prenatal and post-natal [12]. Several lines of evidence have suggested that overexpression of miR-124 inhibited proliferation in medulloblastomas and adult neural precursors [13, 14], and induced neuronal differentiation of both progenitor cells [15] and HeLa cells [16]. However, the molecular mechanisms of miR-124 associated with proliferation and differentiation of NSCs are still unknown.

Notch signaling pathway is an evolutionarily highly conserved morphogenic signaling pathway that plays a crucial role in numerous biological processes, such as cell proliferation, differentiation, maintenance of stem cells, and apoptosis during development and renewal of adult tissues [17, 18]. In mammals, Notch pathway involves four Notch receptors (Notch 1, 2, 3 and 4), and five Notch ligands, including jagged (Jag1 and Jag2) and δ-like canonical Notch ligands Delta-like (DLL1, DLL3 and DLL4), which are reported to be able to regulate the proliferation and self-renewal of NSCs [19, 20]. There is ample evidence demonstrating that Notch pathway, a master regulator of NSCs, plays a key role in the development of nervous system including neuronal differentiation [21].

In the present study, we demonstrated that miR-124 expression was up-regulated and DLL4 expression was down-regulated in NSC differentiation. Moreover, miR-124 was found to target DLL4 and inhibit its expression. Mechanistic analysis revealed that overexpression of miR-124 promoted the proliferation and neural differentiation of NSCs by targeting DDL4 through inactivation of Notch pathways.

## Methods

### NSCs culture, neural differentiation and transfection

This study was approved by the Animal Care and Use Committee of the First Affiliated Hospital of Zhengzhou University. Primary NSCs, obtained from 13.5 days embryos of Wistar rats, were maintained in the growth medium supplemented with 20 ng/mL human epidermal growth factor (hEGF; Gibco, Grand Island, NY, USA), 10 ng/mL basic fibroblast growth factor (bFGF; Gibco), 1% N2 (Gibco), 100 U/mL penicillin/streptomycin (Invitrogen, Carlsbad, CA, USA) at 37 °C in a humidified atmosphere of 5% CO_2_. Neurospheres were digested using 0.25% trypsin into single cell suspension to get clone neurosphere. To induce neural differentiation, neurospheres were seeded onto poly-l-lysine/laminin (invitrogen)-coated coverslips at a density of 2 × 10^4^ cells/coverslip. Subsequently, NSCs were cultured in DMEM-F12 (1:1) differentiation medium supplemented with 1% N2 (Gibco), 2% B27 (invitrogen), 0.5% fetal bovine serum (FBS; Gibco), 20 ng/mL of neurotrophin-3 (NT-3) (PeproTech, Rocky Hill, NJ, USA), 20 ng/mL of brain-derived neurotrophic factor (BDNF) (PeproTech), 10 ng/mL of leukemia inhibitory factor (LIF) (R&D Systems, Inc., Minneapolis, MN, USA), and 2 mM of l-glutamine (Gibco) for 7 days. The differentiation medium was renewed every other day.

To overexpress DLL4, the full-length of DLL4 sequence was synthesized with the sequence released in Genebank (Accession number: NM_019074.2) as a template and inserted to the *Bam*HI and *Xho*I sites of empty pcDNA3.1 vector (invitrogen), named as pcDNA-DLL4. miR-124 mimic (miR-124) (5′-UAA GGC ACG CGG UGA AUG CC-3′), miRNA scrambled control (miR-control) (5′-UUC UCC GAA CGU GUC ACG UTT-3′), miR-124 inhibitor (anti-miR-124) (5′-UAA GGC ACG CGG UGA AUG CC-3′), inhibitor scrambled control (anti-miR-control) (5′-CAG UAC UUU UGU GUA GUA CAA-3′), siRNA targeting DLL4 (si-DLL4), and siRNA scrambed control (si-control) were purchased from the GenePharma (Shanghai, China). The oligonucleotide sequence of si-DLL4 was as follows: 5′-CCT CTC CAA CTG CCC TTC AAT TTCA-3′. NSCs were seeded onto a six-well plate at a density of 2 × 10^5^ cells/well 24 h prior to transfection. Subsequently, NSCs were transfected with 20 nM miRNAs, 40 nM siRNAs or 200 ng pcDNA plasmids using the Lipofectamine 2000 reagent (invitrogen).

### Neurosphere assay

Neurosphere assay was performed to determine the self-renewal ability of NSCs. After introduction with miR-124, miR-control, si-DLL4, si-control, miR-124 + vector, or miR-124 + pcDNA-DLL4, dissociated neurospheres were seeded at a density of 5000 cells in 96-well plates in NSC medium. After 2–3 weeks of incubation, the image fields were captured covered roughly a 3.75 mm^2^ area using an Olympus inverted light microscope (Olympus, Tokyo, Japan) at 200× magnification. The total number of neurospheres with a diameter of > 50 μm was counted and the averaged diameter of neurospheres was measured with ImageJ software (NIH, Bethesda, MD, USA).

### Quantitative real-time PCR

Total RNA was extracted from NSCs using TRIzol reagent (invitrogen) based on the instruction specified by the manufacture. For miR-124 expression detection, aliquots of total RNA (0.5 μg) was reversely transcribed into cDNA using the one-step Primescript miRNA cDNA synthesis kit (Takara, Dalian, China) and RT-PCR was performed using the TaqMan MicroRNA Assay kit (Applied Biosystems Inc., Foster City, CA, USA). For DLL4 mRNA expression evaluation, the first strand of cDNA was synthesized from total RNA using M-MLV reverse transcriptase (Clontech, Palo Alto, CA, USA) and RT-PCR was carried out by the SYBR Green I Real-Time PCR kit (Takara). Real-time PCR was then executed with an ABI7300 Real-Time PCR System (Applied Biosystems Inc.). Relative gene expression levels of miR-124 and DLL4 were calculated using the 2^−ΔΔCt^ method, with U6 and GAPDH as respective internal control.

### Western blot

Protein (50 µg per lane) exacted from NSCs was electrophoresed in 12% SDS-PAGE (Beyotime Institute of Biotechnology, Shanghai, China) and then transferred onto polyvinylidene difluoride membrane (PVDF; Amershame, GE, Buckinghamshire, UK). Following blocked with 5% non-fat milk for 1 h, the membranes were incubated overnight at 4 °C with primary antibodies (Abcam, Cambridge, MA, USA) against DLL4, ki-67, β-tubulin, GAFP, HES1, HEY2, cyclin D1 (CCND1), and β-actin. The membranes were subsequently blotted with HRP-labeled secondary antibody (Santa Cruz Biotech, Santa Cruz, CA, USA) for 1 h at room temperature. The immunoreactive bands were detected using the enhanced chemiluminescence system (Millipore, Billerica, MA, USA).

### Cell proliferation assay

NSCs (5 × 10^4^ cells/well) were seeded into 96-well plates and transfected with miR-control, miR-124, si-DLL4, si-control, miR-124 + vector, or miR-124 + pcDNA-DLL4. MTT analysis was performed to detect cell viability by measuring the optical density at 490 nm at 24, 48, and 72 h posttransfection.

### Luciferase reporter assay

The wild-type or mutant 3′UTR of DLL4 containing the putative miR-124 binding sites was synthesized and inserted into pGL3-luciferase reporter plasmids (Promega, Madison, WI, USA), named as DLL-3′UTR-WT or DLL-3′UTR-MUT. The mutated 3′UTR of DLL4 was generated using QuickChange Site-Directed Mutagenesis kit (Stratagene, La Jolla, CA, USA). Then, NSCs were transfected with DLL-3′UTR-WT or DLL-3′UTR-MUT reporter, pRL-TK (Promega), and miR-124 or miR-control using Lipofectamine 2000 (invitrogen). Cells were harvested 48 h after transfection for luciferase activities measurement with a Dual-Luciferase Reporter Assay system (Promega). Renilla luciferase activity was used as the normalization for firefly luciferase activity.

### Immunocytochemistry

Primary NSCs and their differentiation cells were plated onto coverslips, fixed with 4% paraformaldehyd and permeabilized using 0.3% Triton™X-100 in PBS buffer for 1 h. After blocked with goat serum (10%), cells were incubated with the primary antibodies against GFAP, β-tubulin-III and Nestin (Sigma-Aldrich) at 4 °C overnight, followed by incubation with an Alexa Fluro 568-conjugated secondary antibodies (Santa Cruz Biotech, Santa Cruz, CA, USA) for 1 h. Cell nuclei were counterstained with 1 μg/mL DAPI (Sigma-Aldrich). Slides were analyzed at room temperature on a Zeizz AX10 microscope (Carl Zeiss, Thornwood, NY, USA) using Image J (NIH, Bethesda, Maryland, USA).

### Statistical analysis

All data are presented as mean ± standard deviation (SD) from three independent experiments. All statistical analyses were performed using SPSS 11.7 software (SPSS Inc., Chicago, IL, USA) with two-tailed Student’s *t* test or one-way analysis of variance (ANOVA). For all results, differences were considered as statistically significant when *P* value was less than 0.05.

## Results

### NSCs could self- proliferate and differentiate into neuron and astrocyte

**Fig. 1:**
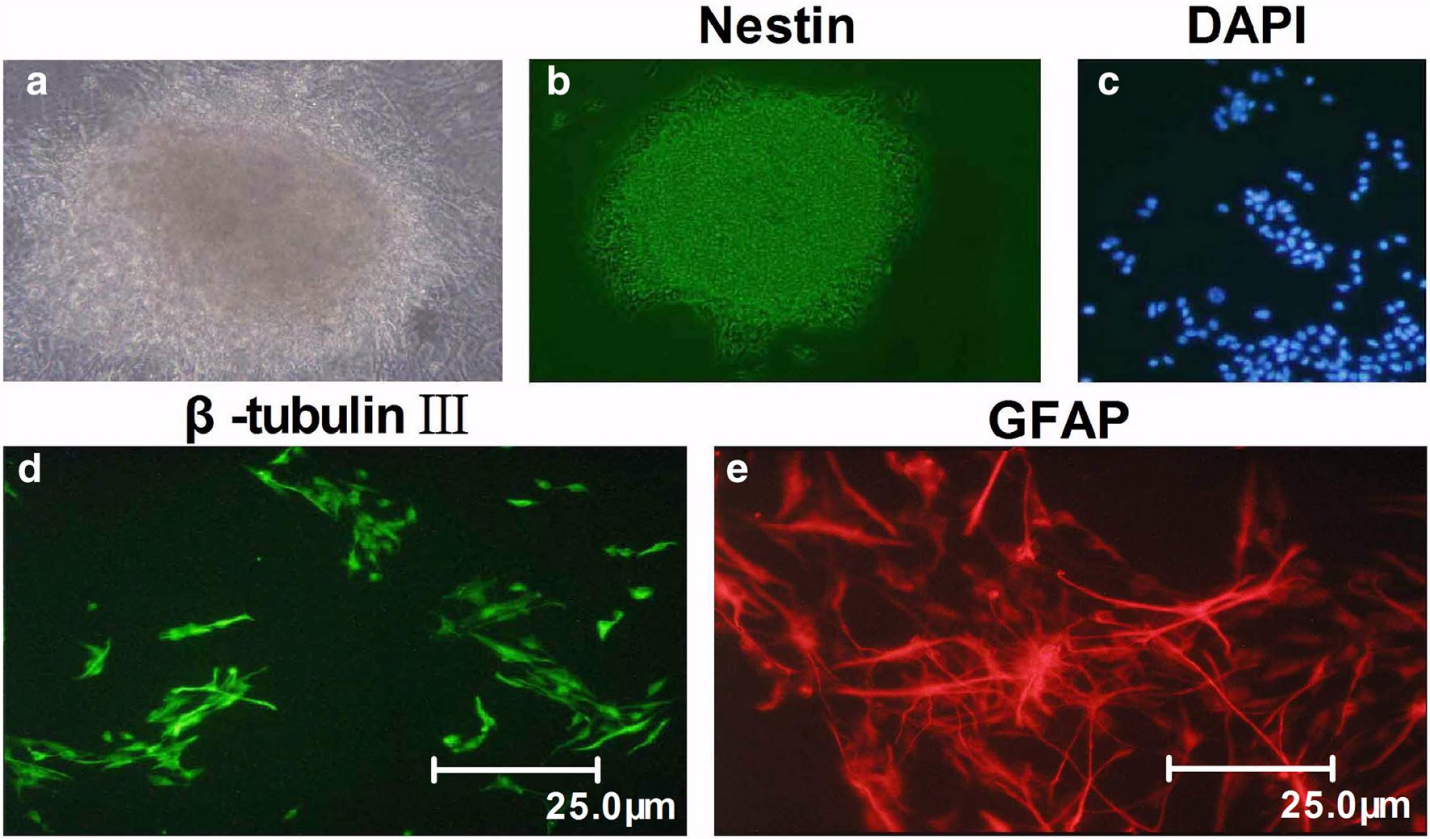
NSCs could self-proliferate and differentiate into neuron and astrocyte. **a** Photomicrographs of representative neurospheres are displayed. **b** Purified NSCs are subjected to immunocytochemical staining with Nestin. **c** Nuclear staining of differentiated cells from NSCs with DAPI. **d** Immunocytochemical staining of purified neurons with β-tubulin-III (×400). **e** Immunocytochemical staining of purified protoplasmic astrocytes with GFAP (×400). n = 3

Isolated primary NSCs could self-proliferate and form neurospheres (Fig. 1a), expressing NSC-specific marker Nestin (Fig. 1b). After removal of EGF and bFGF, these neurospheres differentiated into neurons and astrocytes, as evidenced by the positive expression of neuron-specific marker β-tubulin-III (Fig. 1d) and andastrocyte-specific marker GFAP (Fig. 1e) following DAPI immunostaining (Fig. 1c).

### miR-124 expression was increased and DLL4 was decreased during NSC differentiation

**Fig. 2:**
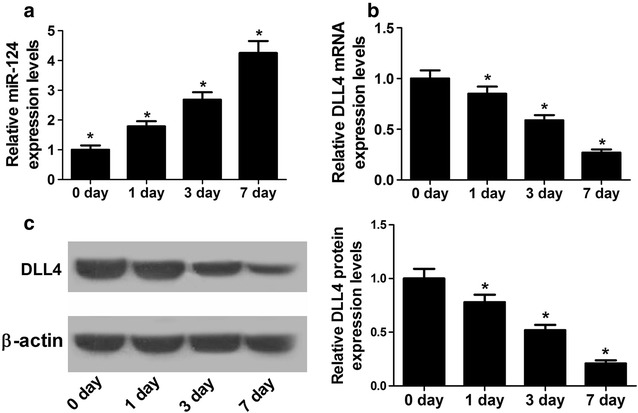
Expressions of miR-124 and DLL4 during NSC differentiation. **a** qRT-PCR analysis of miR-124 expression in NSCs incubated with differentiation medium at day 0, 1, 3, and 7. qRT-PCR and western blot analyses of DLL4 at mRNA (**b**) and protein (**c**) levels in NSCs incubated with differentiation medium at day 0, 1, 3, and 7. *P < 0.05, n = 3

To determine the roles of miR-124 and DLL4 in NSCs differentiation, the expressions of miR-124 and DLL4 at mRNA and protein levels during NSC differentiation were detected by qRT-PCR and western blot. As shown in Fig. 2a, a marked up-regulation of miR-124 expression was observed over time during NSC differentiation. On the contrary, DLL4 expression was down-regulated at both mRNA (Fig. 2b) and protein (Fig. 2c) levels. These data suggested that miR-124 and DLL4 may participate in NSC differentiation.

### DLL4 was a direct target of miR-124 in NSCs

**Fig. 3:**
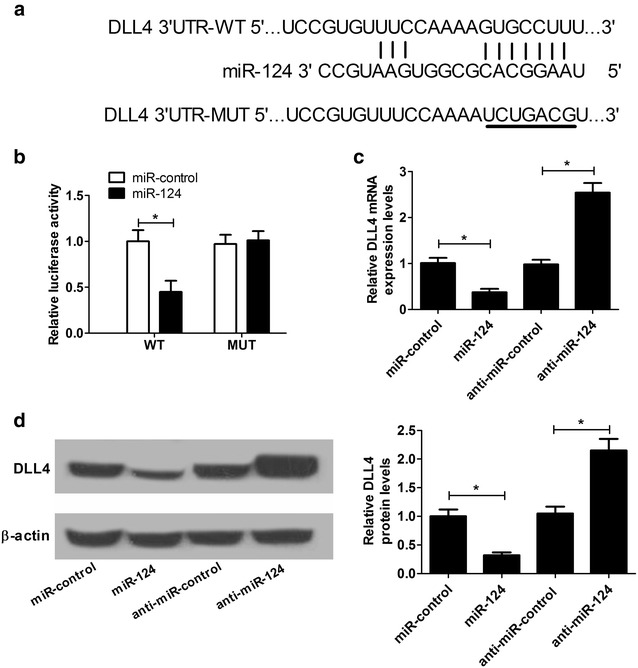
miR-124 directly targeted 3′UTR of DLL4 in NSCs. **a** Online TargetScan website predicted the putative binding sequences of miR-124 in the 3′UTR of DLL4. The sequences of mutated DLL4-3′UTR were also shown. **b** NSCs were cotransfected with the constructed luciferase reporter plasmids (DLL4-3′UTR-WT or DLL4 3′UTR-MUT) and miR-124 or miR-control, and then luciferase reporter assay was performed to detect luciferase activity. The expression of DLL4 at mRNA (**c**) and protein (**d**) levels were assessed by qRT-PCR and western blot in NSC cells transfected with miR-124, anti-miR-124, or matched controls. *P < 0.05, n = 3

TargetScan algorithms were performed to search for the potential targets of miR-124. Bioinformation prediction showed that Jag1, Jag2 and DLL4 were the potential targets of miR-124. Jag1, Jag2 and Notch down-stream effector Sox9 have been identified as the targets of miR-124 by previous studies [14, 22–24]. Therefore, our study focused on DLL4 and firstly investigated whether miR-124 could directly target DLL4. According to the prediction results, a putative binding site for miR-124 was found in the 3′UTR of DLL4 (Fig. 3a). To delineate whether DLL4 was a target of miR-124, the 3′UTR sequences of DLL4 with wild-type or mutated miR-124 binding sites were cloned into luciferase reporter plasmids, and the reporter constructs were cotransfected with miR-124 or miR-control into NSCs. Luciferase reporter assay demonstrated that cotransfection of miR-124 and DLL4-3′UTR-WT markedly restrained the luciferase activity, while cotransfection of miR-124 and DLL4-3′UTR-MUT had no obvious effect on luciferase activity (Fig. 3b). To further verify the target reaction between miR-124 and DLL4, gain- or loss-of-function experiments of miR-124 in NSCs were performed. As shown in Fig. 3c, d, ectopic expression of miR-124 significantly inhibited the expression of DLL4 at mRNA and protein levels in NSCs, while miR-124 inhibition exerted the reverse effects. Collectively, these results demonstrated that miR-124 suppressed DLL4 expression in NSCs by binding to its 3′UTR.

### miR-124 overexpression promoted proliferation and neural differentiation in NSCs

**Fig. 4:**
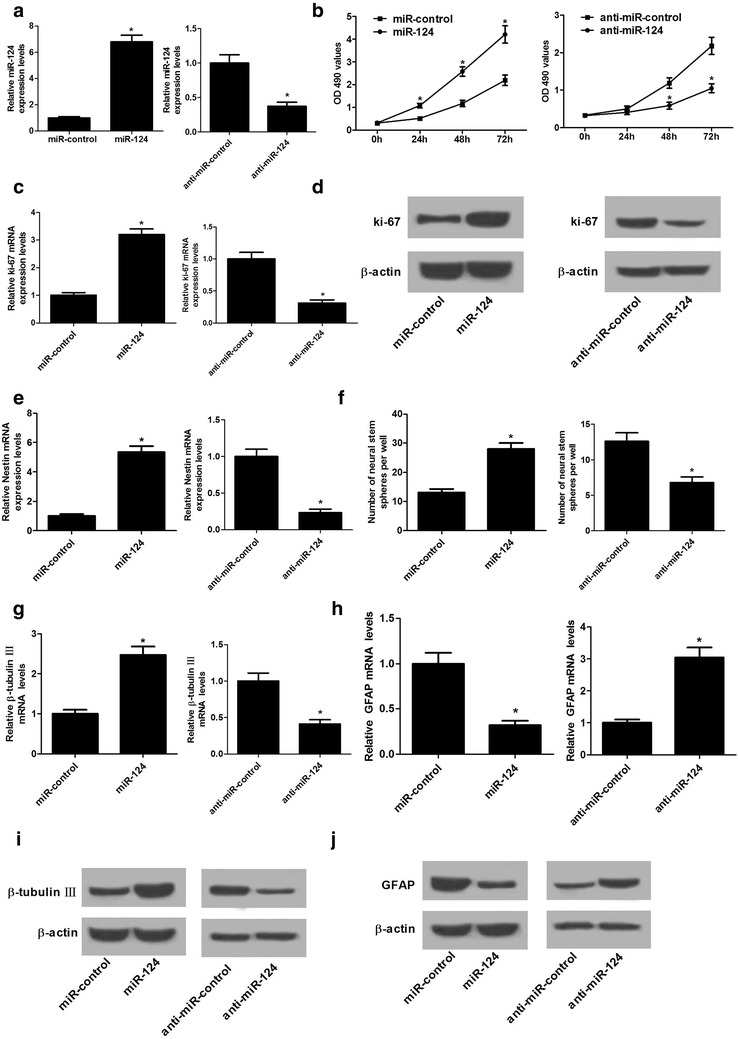
miR-124 overexpression promoted proliferation and neural differentiation in NSCs. NSCs were transfected with miR-124, anti-miR-124, or respective controls. **a** qRT-PCR analysis of miR-124 expression in transfected NSCs. **b** MTT assay was performed to detect cell viability at 0, 24, 48 and 72 h in transfected NSCs. The mRNA (**c**) and protein (**d**) levels of ki-67 in transfected NSCs were evaluated by qRT-PCR and western blot. **e** The mRNA expression of Nestin in transfected NSCs was measured by qRT-PCR. **f** The neurosphere number was counted. The mRNA and protein levels of β-tubulin-III (**g**, **i**) and GFAP (**h**, **j**) in transfected NSCs were evaluated by qRT-PCR and western blot. *P < 0.05, n = 3

To further assess the contribution of miR-124 in NSCs, we evaluated the effects of miR-124 overexpression or downregulation on proliferation and neural differentiation. qRT-PCR analysis demonstrated that miR-124 transfection apparently increased miR-124 expression and anti-miR-124 introduction obviously decreased miR-124 expression in NSCs (Fig. 4a), confirming the transfection efficiency of miR-124 and anti-miR-124. MTT assay revealed that cell growth was significantly improved in NSCs following transfection with miR-124 when compared with miR-control-transfected cells but strikingly suppressed by miR-124 downregulation in NSCs relative to control cells (Fig. 4b). In addition, qRT-PCR and western blot analyses revealed that enforced expression of miR-124 notably elevated and miR-124 inhibition effectively repressed the expression of proliferation marker protein ki-67 at both mRNA (Fig. 4c) and protein (Fig. 4d) levels in NSCs as compared with that in corresponding control group. Consistently, enhanced expression of miR-124 also increased the mRNA expression of NSC-specific marker Nestin and promoted neurospheres formation relative to miR-control group, whereas miR-124 antagomirs exerted the opposite effects (Fig. 4e, f). These data indicated that miR-124 overexpression facilitated NSC proliferation. Also, miR-124 overexpression promoted the expression of neuron-specific marker β-tubulin-III (Fig. 4g, i) and suppressed the expression of astrocyte-specific marker GFAP (Fig. 4h, j) at mRNA and protein levels, while miR-124 suppression showed the reverse effects. These results suggested that miR-124 overexpression promoted neural differentiation in NSCs. Together, these data elucidated that miR-124 overexpression contributed to proliferation and neural differentiation in NSCs.

### DLL4 knockdown promoted proliferation and neural differentiation in NSCs

**Fig. 5:**
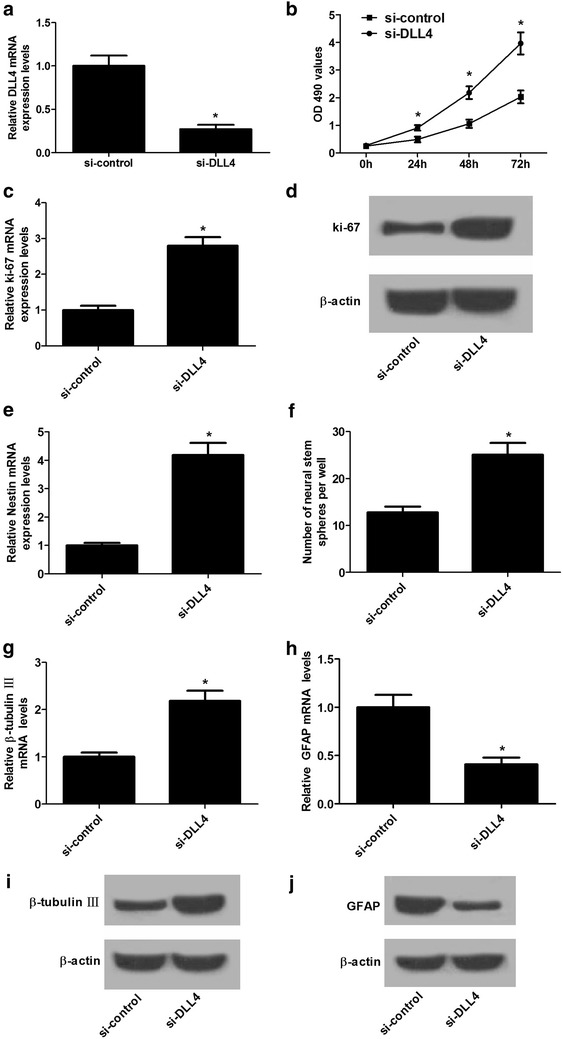
DLL4 knockdown promoted proliferation and neural differentiation in NSCs. NSCs were transfected with si-DLL4 or si-control and cultured for 48 h. **a** miR-124 expression in transfected NSCs was detected by qRT-PCR. **b** MTT assay was conducted to assess cell proliferation in transfected NSCs. The mRNA (**c**) and protein (**d**) levels of ki-67 in transfected NSCs were determined by western blot. **e** The mRNA expression of Nestin in transfected NSCs was analyzed by qRT-PCR. **f** The neurosphere number was counted. The mRNA and protein levels of β-tubulin-III (**g**, **i**) and GFAP (**h**, **j**) in transfected NSCs were examined by qRT-PCR and western blot. *P < 0.05, n = 3

To determine the effect of DLL4 on NSC proliferation and differentiation, loss-of-function approaches were performed in NSCs by transfecting with si-DLL4. As a result, DLL4 expression was significantly decreased in si-DLL4-transfected NSCs compared with si-control group (Fig. 5a). MTT assay implicated that DLL4 silencing effectively promoted NSC proliferation with respect to control group (Fig. 5b). Moreover, it was demonstrated that ki-67 expression at mRNA and protein levels was remarkably elevated by DLL4 knockdown relative to si-control group (Fig. 5c, d). Furthermore, downregulation of DLL4 also enhanced the mRNA expression NSC-specific marker Nestin (Fig. 5e) and facilitated neurospheres formation (Fig. 5f) in NSCs versus si-control group. These results indicated that DLL4 silencing accelerated NSC proliferation. Additionally, we found that DLL4 knockdown markedly increased β-tubulin-III level (Fig. 5g, i) and decreased GAFP level (Fig. 5h, j) in NSCs as compared with si-control group, suggesting that DLL4 knockdown promoted neural differentiation in NSCs.

### miR-124 overexpression promoted proliferation and neural differentiation in NSCs by targeting DLL4

**Fig. 6:**
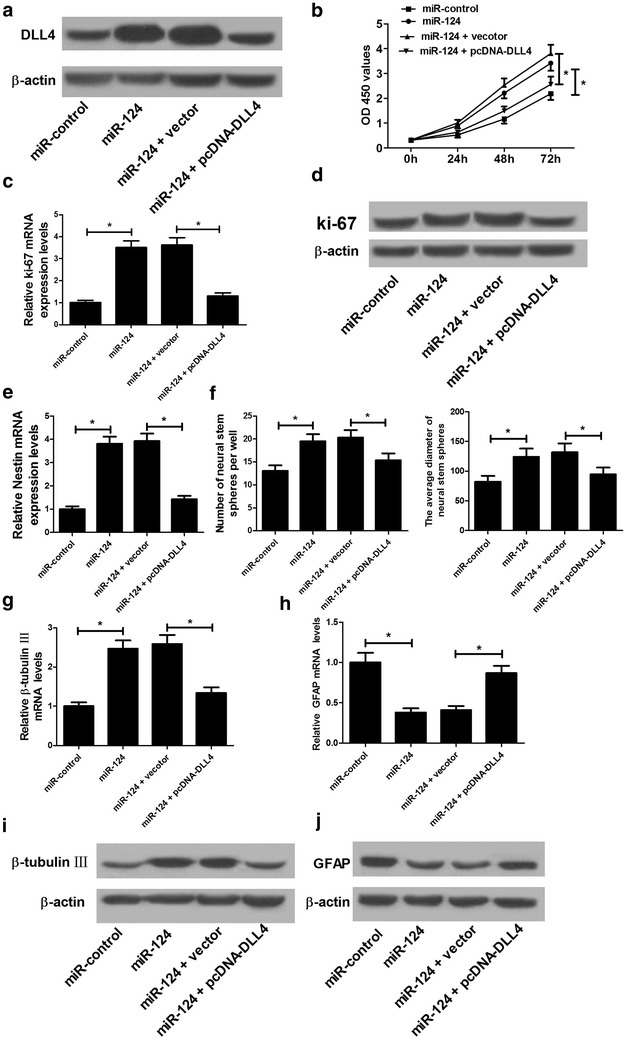
miR-124 overexpression promoted proliferation and neural differentiation in NSCs by targeting DLL4. NSCs were transfected with miR-124, miR-control, miR-124 + vector, or miR-124 + pcDNA-DLL4. **a** Western blot analysis of DLL4 protein level in transfected NSCs. **b** MTT assay was performed to detect cell viability at 0, 24, 48, and 72 h in transfected NSCs. The mRNA (**c**) and protein (**d**) levels of ki-67 in transfected NSCs were determined by qRT-PCR and western blot. **e** The mRNA expression of Nestin in transfected NSCs was examined by qRT-PCR. **f** The number of neurosphere after transfection (diameter > 50 mm) and the averaged neurosphere diameter in different groups were analyzed. The mRNA and protein levels of β-tubulin-III (**g**, **i**) and GFAP (**h**, **j**) in transfected NSCs. *P < 0.05, n = 3

To investigate whether the regulatory effect of miR-124 on NSC proliferation and differentiation was mediated by DLL4, rescue experiments were performed in NSCs by transfecting with miR-124 alone or together with pcDNA-DLL4. Western blot demonstrated that exogenous expression of miR-124 remarkably inhibited DLL4 protein level, while DLL4 overexpression partially restored miR-124-mediated DLL4 expression inhibition (Fig. 6a). MTT assay showed that ectopic expression of DLL4 dramatically attenuated the promotive effect of miR-124 overexpression on cell proliferation in NSC cells (Fig. 6b). We also observed that there was a remarkable increase of ki-67 expression at mRNA (Fig. 6c) and protein (Fig. 6d) levels in miR-124-transfected NSCs, which was partially reversed by DLL4 overexpression. Furthermore, increased expression of DLL4 prominently alleviated the inductive effects of miR-124 on Nestin expression (Fig. 6e), neurospheres formation (Fig. 6f) and β-tubulin-III expression (Fig. 6g, i). Additionally, the decrease of GFAP (Fig. 6h, j) at mRNA and protein levels triggered by miR-124 was partially relieved by exogenous expression of DLL4. Therefore, these findings indicated that DLL4 overexpression alleviated the promotive effects of miR-124 on NSC proliferation and differentiation.

### miR-124 overexpression inhibited the Notch pathway by targeting DLL4

**Fig. 7:**
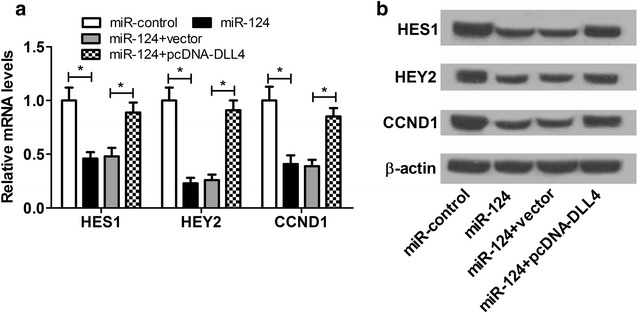
miR-124 overexpression inactivated the Notch pathway by targeting DLL4. **a**, **b** Western blot was performed to determine the protein levels of HES1, HEY2, and CCND1 in NSCs after introduction with miR-124, miR-control, miR-124 + vector, or miR-124 + pcDNA-DLL4. *P < 0.05, n = 3

Since DLL4 was a Notch ligand, we hypothesized that the Notch pathway participated in miR-124-mediated promotion of proliferation and neural differentiation in NSCs. It is known that the Notch signaling affected cell proliferation, survival, and apoptosis by regulating the expressions of target genes [25]. Therefore, we detected the expressions of downstream targets for Notch including HES1, HEY2, and CCND1 in NSCs after introduction with miR-124 alone or together with pcDNA-DLL4. As shown in Fig. 7a, b, forced expression of miR-124 notably repressed the protein levels of HES1, HEY2, and CCND1 in NSCs, whereas overexpression of DLL4 abated the inhibitory effects of miR-124 on the expressions of HES1, HEY2, and CCND1. These data indicated that miR-124 overexpression inactivated the Notch pathway by targeting DLL4.

## Discussion

NSCs, a type of neural progenitor cells, are capable of self-renewing and differentiating into lineages, such as neurons, oligodendrocytes, and astroglia [26]. Nevertheless, the application of NSC-based cell replacement in the therapy of human neurological-related disease remains at its preliminary stage. The major obstacle is to promote NSC proliferation, differentiation, maturation, and functional integration into neural and synaptic networks following NSC transplantation [27]. During CNS development in vertebrates, the maintenance and differentiation of NSCs are tightly regulated by intricate molecular network [28]. In the past few years, increasing evidence have revealed the functional significance of miRNAs in regulating the proliferation and differentiation of NSCs [29]. For instance, elevated expression of miR-1297 [30], miR-765 [31] and miR-381 [32] promote NSC proliferation and differentiation through inhibiting HES1 expression. miR-506-3p modulates NSC proliferation and differentiation partly via transcription factor 3 (TCF3) [33]. miR-125b overexpression inhibits proliferation of neural stem/progenitor cells (NS/PCs) but promotes differentiation and migration by targeting Nestin [8]. In this study, we reporte the role and molecular mechanism of miR-124 during NSC proliferation and differentiation.

Recent studies have revealed that miR-124, the most highly expressed miRNA in the CNS, is closely associated with the development of some CNS diseases. For instance, miR-124 participates in the neural protection of ischemic stroke by activating the PI3K/Akt signaling pathway [34]. Moreover, miR-124-3p, one of the subtypes of miR-124, may play a neuroprotective role in 6-hydroxydopamine-induced cell model of Parkinson’s disease (PD) by targeting annexinA5 (ANXA5) [35]. It was also reported that miR-124 balances the choice between neuronal and astrocyte-specific differentiation in embryonic mouse NSCs by fine-turning a critical epigenetic regulator enhancer of zeste homolog 2 (Ezh2) [36]. Specially, the involvement of miR-124 has been previously documented in neuronal differentiation of mouse inner ear neural stem cells [37]. Inhibition of endogenous miR-124 in NPCs in SVZ suppresses neuronal differentiation, whereas overexpressing miR-124 leads to acquisition of precocious and increased neuron formation [14, 38]. Consistent with the previous study, our findings indicate that miR-124 expression is upregulated during NSC differentiation. Ectopic expression of miR-124 promotes NSC proliferation and increases NSC formation to neurospheres. Moreover, increased expression of miR-124 promotes β-tubulin-III expression and inhibits GFAP expression in NSC cells, suggesting that miR-124 overexpression promotes neural differentiation in NSCs.

The Notch pathway has been reported to play a crucial role in neurodevelopment [21, 39]. Activation of Notch signaling pathway contributes to the maintenance of NSC population, while inactivation of Notch signaling promotes neuronal differentiation of the NSCs [40, 41]. During neural development, the Notch ligands and receptors expressed in CNS are closely involved in the regulation of the proliferation, maintenance, and differentiation of neural progenitor cells [42]. In our study, according to the bioinformatics analysis, DLL4, one of the Notch ligands, was predicted to be a potential target of miR-124. Luciferase reporter assay, qRT-PCR and western blot analysis confirme that miR-124 suppresses the expression of DLL4 by binding to its 3′UTR region in NSCs. DLL4 is found to be downregulated during NSCs differentiation and DLL4 knockdown promotes proliferation and neural differentiation in NSCs. Moreover, elevated expression of DLL4 reverses the promotive effects of miR-124 on NSC proliferation and differentiation. More importantly, transfection of exogenous miR-124 inhibits the Notch pathway in NSCs, which is partially alleviated by DLL4 overexpression. Therefore, these results indicate that miR-124 promotes proliferation and neural differentiation of NSCs by targeting DLL4 via inactivating Notch pathway.

## Conclusions

In conclusion, our study elucidated for the first time that miR-124 promoted the proliferation and neural differentiation of NSCs by targeting DLL4 through suppressing the Notch pathway, contributing to the understanding of molecular mechanism of miR-124 in NSCs. Our study identifies a novel target of miR-124 in NSCs and suggests that manipulating miR-124 might be a novel therapeutic strategy for relevant neurological disorders. However, further studies are still needed to explore whether miR-124 inhibits the Notch pathway by targeting other Notch ligands in NSCs, such as Jag1, Jag2, DLL1, and DLL3, and comprehensively elucidate the molecular mechanism of miR-124 involved in NSC differentiation.

## Abbreviations

NSCs: neural stem cells
DLL4: Delta-like 4
GFAP: glial fibrillary acidic protein
CCND1: cyclin D1
SVZ: subventricular zone
ESC: embryonic stem cell
FBS: fetal bovine serum
PVDF: polyvinylidene difluoride membrane
Ezh2: zeste homolog 2
PD: Parkinson’s disease
ANXA5: annexin A5
TCF3: transcription factor 3
